# Enhanced Operating Temperature Stability of Organic Solar Cells with Metal Oxide Hole Extraction Layer

**DOI:** 10.3390/polym12040992

**Published:** 2020-04-24

**Authors:** Donggu Lee, Junmo Kim, Gyeongtae Park, Hyeong Woo Bae, Myungchan An, Jun Young Kim

**Affiliations:** 1 Realistic Media Research Center, Innovative Technology Research Division, Gumi Electronics & Information Technology Research Institute (GERI), Gumi 39253, Gyeonsangbuk-do, Korea; jmk@geri.re.kr (J.K.); suminyes@geri.re.kr (G.P.); hwbae@geri.re.kr (H.W.B.); amc@geri.re.kr (M.A.); 2 Department of Semiconductor Engineering, Gyeongsang National University, 501 Jinju-daero, Jinju 52828, Gyeongnam, Korea

**Keywords:** polymer solar cell, molybdenum oxide, operating temperature, hole-extraction layer, MoO_3_

## Abstract

Organic solar cells (OSCs) are promising renewable energy sources for replacing fossil fuels. The power conversion efficiency (PCE) of OSCs has increased based on tremendous effort in material and device engineering. Still, the stability of OSC, such as long lifetime, negative temperature coefficient, must be enhanced for commercialization. In this study, we investigated OSC performance at a high operating temperature near 300–420 K, which are typical temperature regions in photovoltaic applications, with a different hole-extraction layer (HEL). The metal oxide-based HEL, MoO_3_, exhibited stable operating properties with a PCE drop rate of −0.13%/°C, as compared to polymeric HEL, PEDOT:PSS (−0.20%/°C). This performance reduction of polymeric HEL originated from the degradation of the interface in contact with PEDOT:PSS, as compared to the robust inorganic metal oxide HEL.

## 1. Introduction

Organic solar cells (OSCs) are promising renewable energy technologies for substituting fossil energy resources because they are thin, light, flexible, and have low manufacturing costs based on the solution process [1,2,3,4,5]. A significant effort in material engineering [6,7,8,9,10], bulk heterojunction morphology control [11,12], and optimization of device structures [13,14,15] has been expended to achieve high power conversion efficiencies (PCEs) in recent decades. Because of these efforts, the PCE of OSCs has reached 17.3% [16], and studies on high efficiency of greater than 14% have been reported regularly [17,18]. However, stability has remained an issue in commercializing OSCs. Recently, a study has been reported to improve the stability through a ternary system by adding 4,4′-Biphenol (BPO) to a polymer:acceptor bulk hetrojunction [19] and an alloy system utilizing two acceptors [20].

In an OSC bulk heterojunction system, the active materials, which produce light absorption, exciton dissociation, and charge collection, are key materials that determine efficiency and stability, although interface layers are also crucial [21,22]. The interface layer of OSC consists of the electron-extraction layer (EEL) and hole-extraction layer (HEL), which extract the generated electrons and holes to the cathode and anode electrode, respectively. In the standard OSC structure, a thin electron extraction material, such as LiF [23], carbon quantum dots that are mixed with ZnO nanorods [24] is used as the EEL, and an n-type metal oxide, such as ZnO, is used as an optical spacer and EEL [25]. Polymeric materials, such as poly(3,4-ethylenedioxythiophene): polystyrene sulfonate (PEDOT:PSS), are commonly used for the HEL of OSCs and perovskite solar cells (PSCs) [26]. However, PEDOT: PSS has acidic properties that degrade the properties of indium tin oxide (ITO) electrodes and is not stable to moisture, adversely affecting the PCE and stability of the OSCs [27]. Consequently, instead of PEDOT:PSS, new polymeric materials, such as PANI:PSS [28], a new sulfonated polyaniline derivative containing thiol groups (SPAN(SH)) that are mixed with gold nanoparticles [29], have been recently applied as HELs. Metal oxides, such as MoO_3_ [30], V_2_O_5_ [31], and WO_x_ [32], are also suitable candidates for HELs.

Several studies have been reported on the characteristics of OSCs at high operating temperatures on solar cells [33,34,35]. However, in order to improve the stability of OSC, further studies are needed on high-temperature operation at 27 (RT, room temperature)—147 °C, which is common in photovoltaic applications.

The temperature dependency of the open-circuit voltage (*V*_OC_) of OSCs is expected from the conventional PN junction solar cells [33]; V_OC_ is expressed as

(1) $$V_{OC}=\frac{nkT}{q}\ln\left(\frac{I_{SC}}{I_{0}}+1\right)$$

where *n* is the diode ideality factor, *k* is the Boltzmann constant, *T* is temperature, *q* is the elementary charge, *I*_SC_ is the short-circuit current, and *I*_0_ is the reverse saturation current.

For a geometrically simple model of Shockley, I_0_ is expressed as

(2) $$I_{0}=qN_{v}N_{c}\left[exp\left(-\frac{E_{g}}{kT}\right)\right]\cdot \left(\frac{L_{n}}{n_{n}\tau_{n}}+\frac{L_{p}}{n_{p}\tau_{p}}\right)$$

where *N*_V_ and *N*_C_ are the effective densities of states in the valence band and conduction band, *E*_g_ is the bandgap of the semiconductor, *L*_n_ and *L*_p_ are diffusion lengths of electrons and holes, *n*_n_ and *n*_p_ are the carrier densities of electrons and holes, and *τ*_n_ and *τ*_p_ are the lifetimes of the electrons and holes, respectively.

Therefore, *I*_SC_ ≫ *I*o, and by inserting Equation (2) into Equation (1), *V*_OC_ is given as

(3) $$V_{OC}=\frac{nE_{g}}{q}-\frac{nkT}{q}\ln\left[\frac{1}{I_{SC}}\cdot qN_{v}N_{c}\cdot \left(\frac{L_{n}}{n_{n}\tau_{n}}+\frac{L_{p}}{n_{p}\tau_{p}}\right)\right]$$

which denotes a decrease in *V*_OC_ as temperature increases.

I_SC_ is difficult to apply with conventional PN junction solar cells, because organic semiconducting materials have electrical properties that differ with temperature, in contrast to inorganic materials, such as increasing mobility with increasing temperature [36,37]. Furthermore, the temperature characteristics of I_SC_ are dependent on the active material. For instance, polymer solar cells with MDMO-PPV active materials exhibited a monotonic increase of I_SC_ as temperature increased until 65 °C, and were then saturated [33]. However, the OSC that was based on P3HT:PCBM had the highest I_SC_ value at RT and decreased as the temperature increased [34].

Fill factor (FF) and PCEs are also challenging to predict according to the operating temperature, because a complex mechanism combining charge carrier generation, recombination, transport, and collection to the electrode determines these parameters. Therefore, a systematic study is needed to understand the performance of solar cells at high operating temperatures near RT–127 °C.

In this study, we focused on the effect of the HEL on high-temperature operation (room temperature [RT] to 147 °C) of OSC with metal oxide-based (MoO_3_) and polymer-based (PEDOT:PSS) HELs. The OSC with MoO_3_ exhibited a small efficiency drop coefficient of 0.13%/°C when compared to PEDOT:PSS (0.20%/°C). This reduction in efficiency is primarily attributed to a decrease in the short-circuit current density (*J*_SC_), which is caused by a decrease in the surface recombination velocity and the degradation of the polymer HEL. A rapid rise of R_S_ above 87 °C denotes this degradation.

## 2. Materials and Methods

All of the devices were prepared on indium-tin-oxide (ITO) coated glass with ~150 nm thickness and ~20 Ω/square sheet resistance. The ITO-coated substrates were cleaned using isopropyl alcohol, de-ionized water, acetone, and methanol in an ultrasonic bath at air condition and then dried in a vacuum oven. The MoO_3_ as a HEL with a 10 nm thickness was thermal-evaporated under a vacuum condition of ~10^−6^ Torr. The PEDOT:PSS as an HEL was spin-coated on ITO substrates with a spin rate of 4000 rpm for 30 sec at air condition and then dried at 120 °C for 30 min. in the vacuum oven (thickness of PEDOT:PSS film: 40 nm). Subsequently, the 3 wt % solution of P3HT (Rieke metals, 4002-E):PCBM (Nano-C) (1:0.8 by weight.) dissolved in monochlorobenzene was spin-coated on the HEL in the glove box filled with Ar gas and the thickness of P3HT:PCBM film is ~120 nm. After spin-coating the photoactive layer, LiF (thickness: 0.5 nm) as the EEL and Al (thickness: 100 nm) as the cathode were thermal-evaporated under a vacuum condition of ~10^−6^ Torr. All of the devices were post-annealed at 150 °C for 30 min. in the glove box filled with Ar gas and then encapsulated with a UV sealant (Nagase ChemteX Corp, XNR 5570-B1) with a glass cap. The active area of the fabricated solar cell was 0.09 cm^2^.

The current density-voltage (J-V) curves under 1 to 100 mW/cm^2^ illumination from a solar simulator (Newport 91160 A, AM 1.5 G with a variable neutral density filter) were measured in a vacuum closed-cycle refrigerator while using a Keithley 237 source measuring unit. The temperature controller (Lake Shore Cryotronics 331) is N_2_-based and it can measure RT at 27 °C and a high temperature up to 147 °C. The measurement started at 27 °C, and the temperature was increased up to 147 °C by raising the temperature by 30 °C intervals (RT → 57 °C → 87 °C → 117 °C → 147 °C). The measurement was delayed for 1 h after setting the temperature to ensure temperature stability.

## 3. Results and Discussion

**Figure 1:**
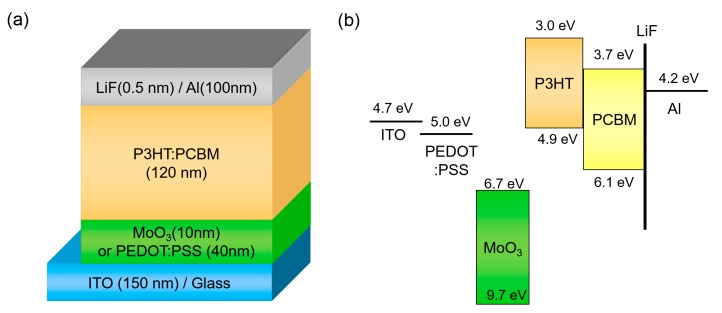
(**a**) Device structure and (**b**) energy band diagram of organic solar cell (OSC) with different hole-extraction layers (HELs).

We adopted a conventional OSC structure, which consists of ITO/HEL/P3HT: PCBM/LiF/Al, to investigate the effect of operating temperature. Figure 1 illustrates the device structure and energy band diagram of OSC. PEDOT:PSS is the most conventional HEL in OSCs, and MoO_3_ is representative of metal-oxide hole injection materials in organic electronics. When compared to PEDOT:PSS, which is a highly-conductive polymer material with a high work function of 5.0 eV [38], MoO_3_ has a deep-lying conduction band energy level and it can extract holes strongly in P3HT [39].

In the case of P3HT:PCBM solar cells, the bulk heterojunction morphology is altered by the post-annealing temperature. As a result, the mobility and JSC, FF, and PCE of the OSC are significantly improved [25,37]. Therefore, the temperature-dependent J-V characteristics of all devices were acquired after post-annealing at 150 °C in order to evaluate the performance of OSCs with optimized bulk heterojunction morphology. The surface topography of P3HT:PCBM films exhibited similar morphology, regardless of the type of HEL (See, Figure S1 in Supplementary Materials).

**Figure 2:**
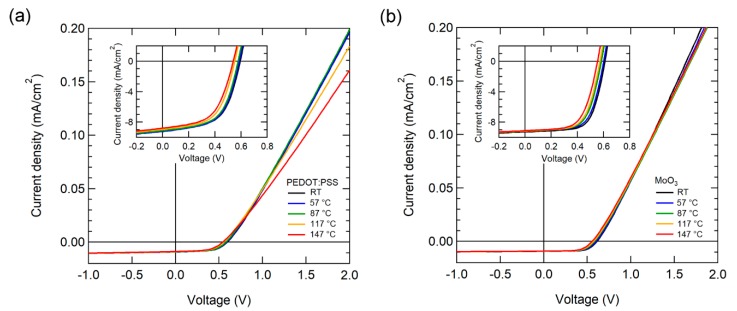
Current density (J)-voltage (V) characteristics of OSCs with various temperatures using (**a**) PEDOT:PSS and (**b**) MoO_3_ as HEL under 1-sun illumination. The inset illustrates the enlarged fourth quadrant area of the J-V curve.

The J-V characteristics of OSCs were measured by gradually increasing the temperature from RT to 147 °C. Figure 2 illustrates the temperature-dependent J-V characteristics of OSCs while using PEDOT:PSS and MoO_3_ as HELs under AM 1.5 G 1-sun illumination. The current density of the OSC with PEDOT:PSS decreased noticeably over 87 °C, as depicted in Figure 2a. Moreover, the OSC with MoO_3_ exhibited more stable J-V characteristics with a small decrease in current density.

For the OSC performance parameter at RT, the device with PEDOT: PSS had a *J*_SC_ of 9.65 mA/cm^2^, *V*_OC_ of 0.59 V, FF of 57.1%, and PCE of 3.27%. Meanwhile, the device with MoO_3_ had a *J*_SC_ of 9.29 mA/cm^2^, *V*_OC_ of 0.61 V, FF of 66.2%, and PCE of 3.73%. The OSC with MoO_3_ exhibited superior *V*_OC_, FF, and PCE characteristics when compared to PEDOT: PSS. Table 1 presented the performance parameters of the higher operating temperature.

**Figure 3:**
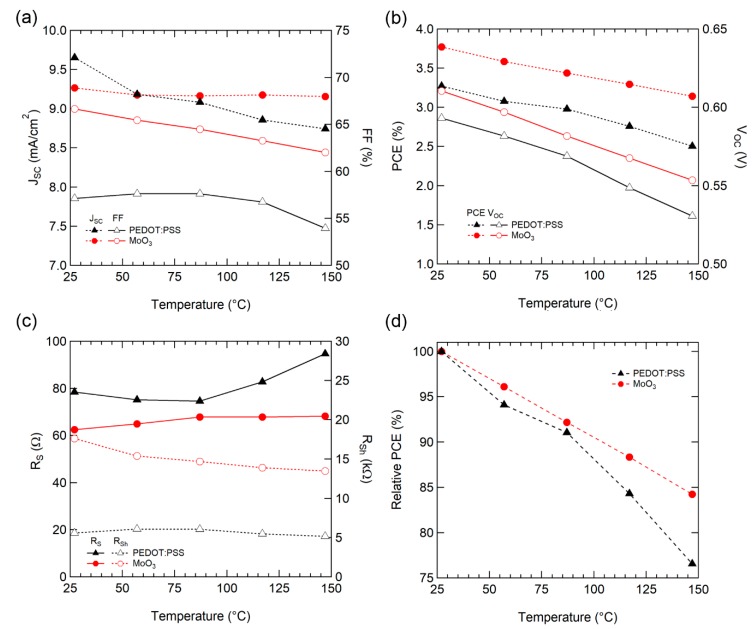
(**a**) *J*_SC_ and fill factor (FF), (**b**) power conversion efficiency (PCE) and *V*_OC_, (**c**) R_S_ and R_Sh_, and (**d**) Normalized PCE characteristics of OSCs with various temperatures.

The performance parameters of the solar cells were extracted for each temperature to analyze the detailed effect of operating temperature. For *J*_SC_ (Figure 3a), PEDOT: PSS tended to decrease as the temperature increased, whereas MoO_3_ exhibited a small J_SC_ loss as the temperature increased. The electrical mobility [37,40] and conductivity [41] of P3HT:PCBM films increased as temperature increased due to thermal-assisted hopping, but decreased with increasing temperature for *J*sc, as in the previous study [34].

For *V*_OC_ (Figure 3b), as expected from Equation (3), the temperature characteristics of V_OC_ are comprised of parameters from the OSC active materials. Consequently, both types of HELs decreased similarly with a slope of −0.53 mV/°C for PEDOT:PSS and −0.48 mV/°C for MoO_3_.

FF is affected by various material properties, such as charge carrier transport (mobility) and recombination (lifetime, recombination), carrier collection into the electrodes (interface between active/buffer layer and electrodes). PEDOT:PSS maintained similar values up to 87 °C and then decreased; however, the OSC with MoO_3_ decreased steadily, as depicted in Figure 3a.

For a detailed analysis of FF, series resistance (R_S_, from high voltage region [>1 V]) and shunt resistance (R_Sh_, near the short circuit condition [~0 V]) were extracted from the IV curve (Figure 3c). For MoO_3_, R_S_ increased and R_Sh_ decreased steadily as the temperature increased, which is consistent with the steady decline in FF. For PEDOT: PSS, a noticeable increase of R_S_ was observed above 87 °C, and R_Sh_ had a similar value throughout the entire temperature range. This increase in R_S_ causes a drop of FF above 87 °C. The charge carrier mobility in PEDOT:PSS [41] and P3HT:PCBM [37,40] films increase as the temperature rises. This increase of R_S_ implies a degradation of the interface in contact with PEDOT: PSS.

Consequently, the normalized conversion efficiency of the OSC with MoO_3_ exhibited enhanced operating temperature stability with a drop factor of −0.13%/°C, when compared to PEDOT:PSS (−0.20%/°C), as illustrated in Figure 3d. Our OSCs display superior temperature stability as compared to a typical single crystal silicon solar cell (−0.40%/°C) [42].

**Figure 4:**
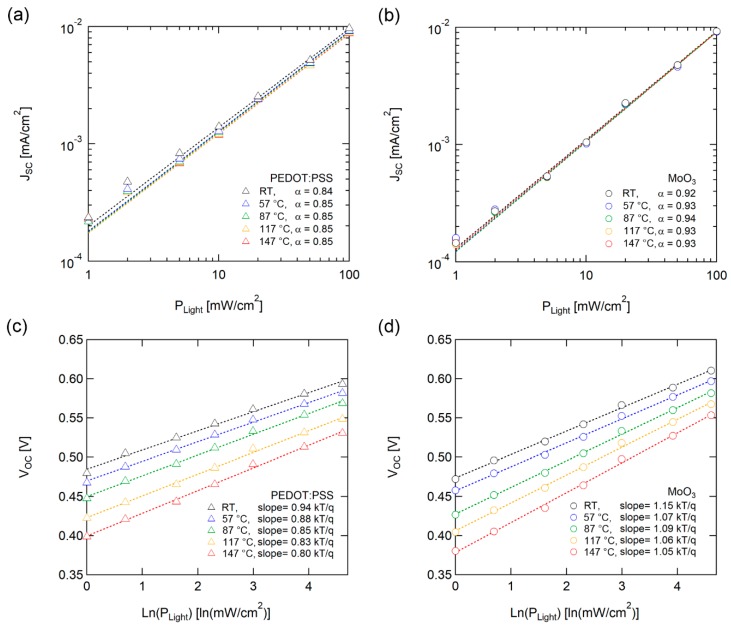
Light intensity dependence of (**a, b**) *J*_SC_ and (**c, d**) *V*_OC_ with PEDOT:PSS (left) and MoO_3_ (right) as HELs. Dotted lines denote fitting lines. *J*sc was fitted according to *J*_SC_ ~ (P_light_)^α^, and *V*_OC_ was fitted logarithmically with light intensity (ln(P_light_)). The fitting results are below each set of curves.

We performed incident light intensity-dependent J-V measurements to analyze the recombination characteristics of the solar cells. Figure 4a and 4b illustrate the light intensity (P_light_) dependence of J_SC_ with various temperatures. J_SC_ follows power-law dependence with incident light intensity, J_SC_ ~ (P_light_)^α^. The *α* correlated with the losses caused by bimolecular recombination. For weak bimolecular recombination, in which monomolecular recombination is the dominant mechanism, α is close to 1. Meanwhile, α is close to 0.5 if bimolecular recombination is dominant [43]. OSCs with both PEDOT:PSS and MoO_3_ demonstrated a lack of temperature dependence on α, suggesting that the dominant recombination mechanism is not changed during high-temperature operation.

Figure 4c and 4d display light intensity dependence between *V*_OC_ and various temperatures. The light intensity dependence of *V*_OC_ provides supplemental and additional information regarding recombination processes from the J_SC-_P_light_ analysis. The *V*_OC_ of solar cells exhibited dependency logarithmically with light intensity (ln(P_Light_)), and the slope of thermal voltage (*kT*/*q*) correlated with the recombination mechanism with trap states [44,45]. The OSC with MoO_3_ did not exhibit noticeable temperature dependence of the slope, but the OSC with PEDOT:PSS decreased under unity as the temperature increased. J. Cheng et al. reported a large injection barrier or finite surface recombination velocity, block charge extraction, and charge accumulation, resulting in modified built-in potential and degrading the performance of OSCs, with a decrease in slopes under unity [46]. The slope of V_OC_-ln (P_light_) under unity of OSC with PEDOT:PSS indicates a decrease in surface recombination velocity caused by the degradation of the interface with PEDOT:PSS as the temperature increased, as illustrated in Figure 4c. In contrast, MoO_3_ had robust interface characteristics based on the high thermal stability of inorganic material.

## 4. Conclusions

We investigated the effect of HELs on OSC performance at high operating temperatures. The metal oxide-based HEL, MoO_3_, exhibited stable operating properties with a PCE drop rate of −0.13%/°C, when compared to polymeric HEL, PEDOT:PSS (−0.20%/°C). The performance drop of the OSC with PEDOT:PSS revealed an increase in R_S_ and a decrease in surface recombination velocity. This implies a degradation of the interface in contact with PEDOT:PSS. In contrast, the MoO_3_ HEL maintains interface properties at high-temperature operation, thus resulting in stable operation.

The results of this study demonstrate that metal oxide-based HELs can provide robust high-temperature stability of OSCs with other high-efficiency active materials. Furthermore, this metal oxide-based HEL is a suitable candidate that can improve the stability of organic electronics, driving in high-temperature environments, such as OLED, not only OPV.

## Figures and Tables

**Table 1:** Performance parameters of OSCs as a function of operating temperature using PEDOT:PSS and MoO_3_ as HEL under 1-sun illumination.

HEL	Temp. (°C)	*J*_SC_ (mA/cm^2^)	*V*_OC_ (V)	FF (%)	PCE (%)
PEDOT:PSS	27 (RT)	9.65	0.59	57.1	3.27
	57	9.18	0.58	57.6	3.08
	87	9.08	0.57	57.6	2.98
	117	8.86	0.55	56.7	2.76
	147	8.75	0.53	54.0	2.50
MoO_3_	27 (RT)	9.27	0.61	66.7	3.77
	57	9.17	0.60	65.5	3.58
	87	9.17	0.58	64.5	3.44
	117	9.18	0.57	63.3	3.30
	147	9.15	0.55	62.0	3.14

## Supplementary Materials

The following are available online at https://www.mdpi.com/2073-4360/12/4/992/s1, Figure S1: AFM topography of P3HT:PCBM films on (a) PEDOT:PSS and (b) MoO_3_.
