# Supplementary material for: Enhanced Operating Temperature Stability of Organic Solar Cells with Metal Oxide Hole Extraction Layer
Source: Polymers (Basel). 2020 Apr 24;12(4):992. doi: 10.3390/polym12040992 (PMC7240709; doi:10.3390/polym12040992)
Supplement: Supplementary file 1 [file polymers-12-00992-s001.zip › polymers-787188-supplementary.pdf]

polymers

MDPI

Supplementary Materials

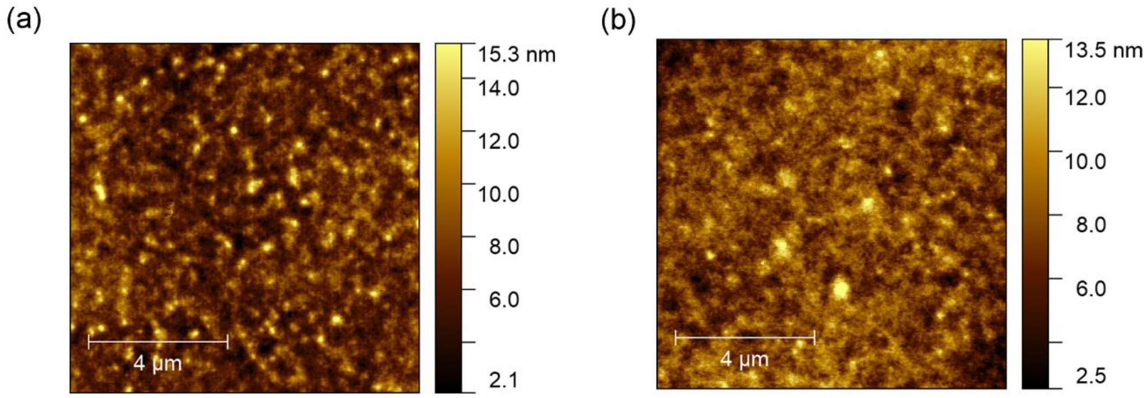

Figure S1. AFM topography of P3HT:PCBM films on (**a**) PEDOT:PSS and (**b**) MoO_3. Root-mean-square (RMS) roughness of P3HT:PCBM film on PEDOT:PSS and MoO_3 were 1.92 nm and 1.58 nm, respectively.

© 2020 by the authors. Submitted for possible open access publication under the terms and conditions of the Creative Commons Attribution (CC BY) license (http://creativecommons.org/licenses/by/4.0/).
